# Incidence of airway complications associated with deep extubation in adults

**DOI:** 10.1186/s12871-020-01191-8

**Published:** 2020-10-29

**Authors:** Jeremy Juang, Martha Cordoba, Alex Ciaramella, Mark Xiao, Jeremy Goldfarb, Jorge Enrique Bayter, Alvaro Andres Macias

**Affiliations:** 1 Department of Anesthesiology, Massachusetts Eye and Ear, 243 Charles St, Boston, MA 02114 USA; 2 Harvard Medical School, Boston, MA 20114 USA; 3 Clinica El Pinar, Km 2 Anillo vial Floridablanca – Girón, Ecoparque Empresarial Natura Torre 2 piso 1 y 2, Piedecuesta, Colombia

**Keywords:** Tracheal extubation, Deep extubation, Airway, Anesthesia, Ambulatory surgery, Emergence, Complications, Adult, Volatile anesthetics

## Abstract

**Background:**

Endotracheal extubation is the most crucial step during emergence from general anesthesia and is usually carried out when patients are awake with return of airway reflexes. Alternatively, extubations can also be accomplished while patients are deeply anesthetized, a technique known as “deep extubation”, in order to provide a “smooth” emergence from anesthesia. Deep extubation is seldomly performed in adults, even in appropriate circumstances, likely due to concerns for potential respiratory complications and limited research supporting its safety. It is in this context that we designed our prospective study to understand the factors that contribute to the success or failure of deep extubation in adults.

**Methods:**

In this prospective observational study, 300 patients, age ≥ 18, American Society of Anesthesiologists Physical Status (ASA PS) Classification I - III, who underwent head-and-neck and ocular surgeries. Patients’ demographic, comorbidity, airway assessment, O_2_ saturation, end tidal CO_2_ levels, time to exit OR, time to eye opening, and respiratory complications after deep extubation in the OR were analyzed.

**Results:**

Forty (13%) out of 300 patients had at least one complication in the OR, as defined by persistent coughing, desaturation SpO_2_ < 90% for longer than 10s, laryngospasm, stridor, bronchospasm and reintubation. When comparing the complication group to the no complication group, the patients in the complication group had significantly higher BMI (30 vs 26), lower O_2_ saturation pre and post extubation, and longer time from end of surgery to out of OR (*p* < 0.05).

**Conclusions:**

The complication rate during deep extubation in adults was relatively low compared to published reports in the literature and all easily reversible. BMI is possibly an important determinant in the success of deep extubation.

## Background

Endotracheal extubation is the final and arguably the most crucial step during emergence from general anesthesia (GA). Normally, it is carried out when patients are awake with return of airway reflexes. However, extubations can also be accomplished while patients are deeply anesthetized but maintaining spontaneous breathing, a technique known as “deep extubation”. Deep extubation is frequently performed in the setting of eye surgery as well as head and neck surgery. The intention is to minimize bucking and limit increase in intraocular and intracranial pressure [1–4].

When surveyed, even in appropriate clinical situations, many anesthesiologists are still reluctant to perform deep extubation in adults because of concerns for potential respiratory complications [5]. This apprehension may be unfounded as most published experiences (and reported complications) center around pediatric patients [6–9] and not adult patients. To our knowledge, there have only been a couple of adult deep extubation studies, with around 30 patients in each arm, comparing respiratory complications in patients deeply extubated after inhaled anesthetics with and without adjuvants [10, 11]. More robust data in a larger adult population are needed to inform clinical practice.

Therefore, in this prospective observational cohort study, we set out to assess the rate of respiratory complications after deep extubation in a larger sample size of 300 adult patients undergoing ocular and head and neck surgery. Our goal was to determine if there are intraoperative factors that may influence the success of deep extubations.

## Methods

### Study population

This single arm, unblinded, observational study was approved by the Institutional Review Board (IRB) of Massachusetts Eye and Ear Infirmary, Boston, Massachusetts (#1047249). The study was conducted in accordance with all rules and regulations laid out by the IRB and human studies committee. A waiver of written informed consent was obtained for this study. This study was registered at Clinicaltrials.gov (NCT04557683).

Patients greater than 18 years of age at the time of surgery and selected by the anesthesiologist as a candidate for deep extubation were enrolled in this study without specific exclusion criterion. All patients were evaluated by the preoperative anesthesia staff prior to surgery and a detailed preoperative note detailing vital signs, health history, and airway assessment (Mallampati score I-IV, neck ROM, TM distance, mouth opening, and artificial airway, facial hair, dental exam) was documented in the electronic medical record. Over the course of six months, 300 patients were enrolled in this observational study. Each day during this six-month period, a research coordinator would report to the main operating room and determine the possible candidates for the day based on age and anesthetic plan. Towards the end of each surgery the research coordinator would ask each anesthesiologist utilizing inhalation anesthetics about the extubation plan. If the anesthesiologist selects the patient for deep extubation, the patient would be followed from the end of surgery to Post Anesthesia Care Unit (PACU) for data collection. The deep extubation technique was the only controlled procedural variable among our patient cohort; other anesthesia procedural variables were selected at the provider’s discretion.

### Anesthetic management

At the end of the case, the fraction of inspired oxygen (FiO_2_) was increased to 100% and the end inspired concentration of inhaled anesthetic was adjusted to be at least 1 Minimum Alveolar Concentration (MAC) or higher if needed. The depth of anesthesia was considered adequate clinically when the patient was spontaneously breathing with a regular pattern, at a MAC of 1 or higher, and if the patient did not exhibit any response to suctioning and to deflation and reinflation of the endotracheal tube cuff. Before extubation, an oral airway was placed in all the patients, and jaw thrust was applied if needed after extubation. The oral airway was removed, either in the operating room by anesthesia provider or in PACU by trained PACU nursing staff with 1-to-1 nurse to patient ratio under the supervision of an anesthesiologist, when the patient regained airway reflexes. Patients were administered oxygen at 6 L/min, via a face mask; supplemental oxygen was discontinued in PACU as per usual recovery room management.

### Statistical analysis

For comparison, patients were classified into two groups: those without respiratory complications to those with respiratory complications as defined by persistent coughing, desaturation measured by saturation of peripheral oxygen (SpO_2_) by pulse oximetry of less than 90% for longer than 10s, laryngospasm, stridor, bronchospasm, and reintubation. Patient demographics, baseline characteristics, procedures, intubation notes, and intraoperative variables were obtained from the electronic medical records and analyzed. Statistical analysis and graphs were performed and presented using Prism 8.4.2 (GraphPad Software Inc., La Jolla, CA). The normality of the distribution of continuous variables was assessed using the Shapiro-Wilk normality test. Mann-Whitney tests were used to compare continuous variables among groups. A 2-tailed *P*-value less than 0.05 was considered significant. Fisher’s exact test was used to compare categorical variables among groups. Continuous variables are presented as median with interquartile ranges (q1-q3), while categorical variables are summarized using frequencies and percentages.

## Results

**Fig. 1:**
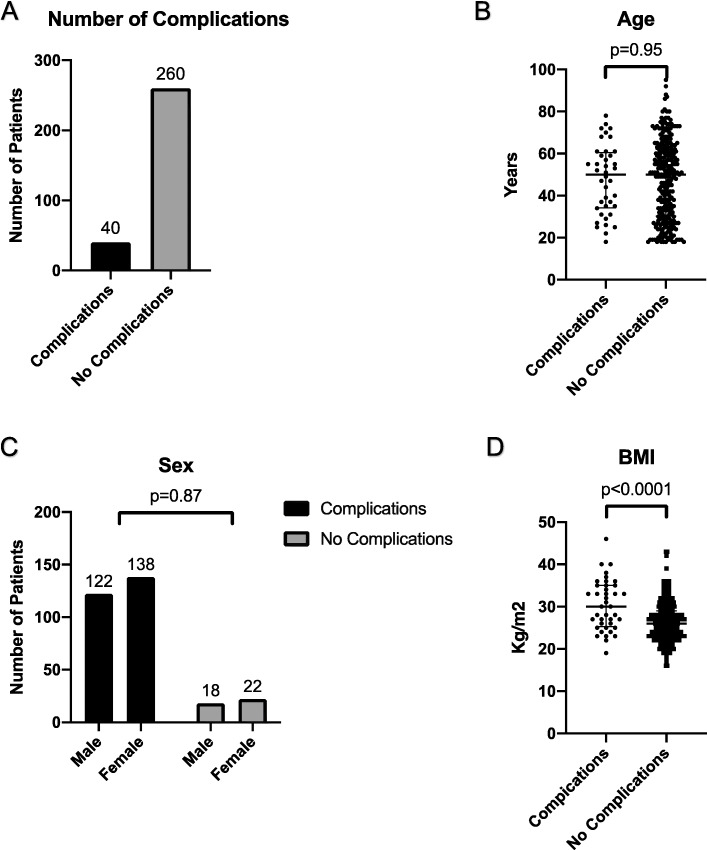
Number of patients with at least one complication^*^ in the OR after deep extubation (**a**) and comparison of patient demographics between complications and no complications group by (**b**) Age, (**c**) BMI, and (**d**) Sex. ^*^Complications include desaturation SpO2 < 90% for longer than 10s, persistent cough, laryngospasm, stridor, bronchospasm, and reintubation

A total of 300 adult patients were recruited for the study. Among them, 40 (13%) patients had at least one complication in the OR post deep extubation that included persistent coughing, desaturation SpO_2_ < 90% for longer than 10s, sore throat, laryngospasm, stridor, bronchospasm (Fig. 1a). None of the 300 patients required re-intubation.

When comparing patient’s demographic of the complications group to the no complications group, there were no differences in patient age (50.0(34.4–60.5) vs 50.0(30.3–52.0), *p* = 0.9506) (Fig. 1b) and sex (Fig. 1c). In contrast, patients in the complications group had significantly higher BMI (30.0(25.3–35.0) vs 26.0(23.0–29.0), *p* < 0.0001) when compared to the no complications group (Fig. 1d).

**Fig. 2:**
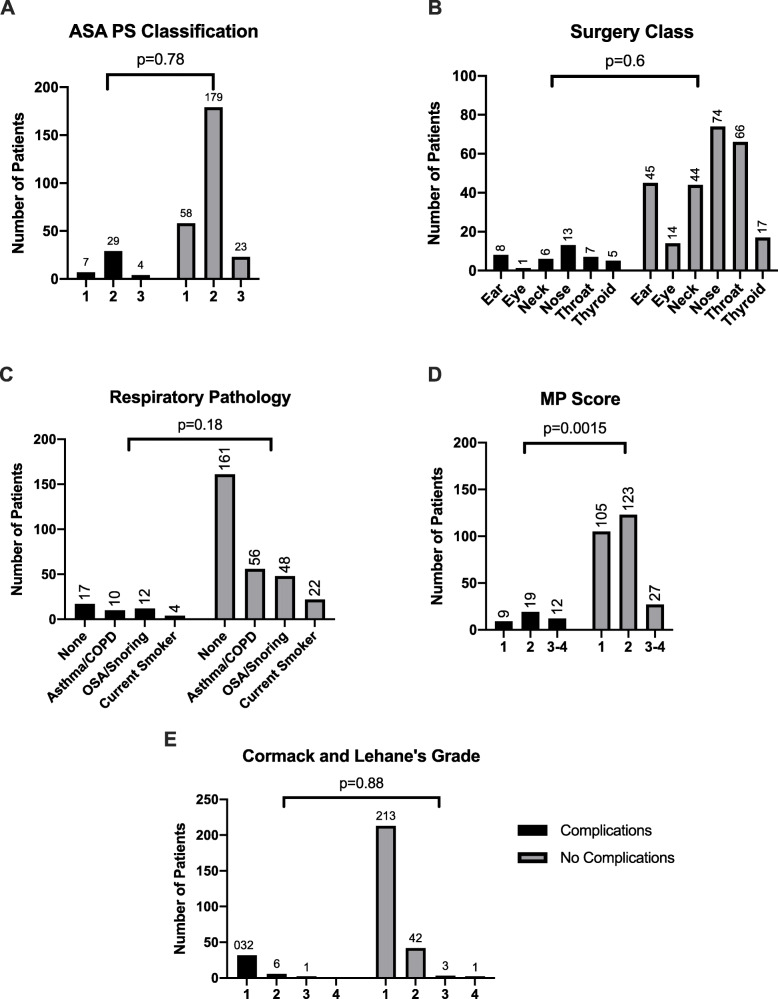
Comparison of patients and intraoperative characteristics between complications versus no complications groups by **a** ASA PS Classification, **b** Surgery Class, **c** Respiratory Pathology, **d** Mallampati (MP) Score, **e** Cormack-Lehane Grade

We observed no significant difference in patient ASA PS classification or type of surgery class (ear, eye, neck, nose, throat, thyroid) (Fig. 2 a&b). Furthermore, there were no significant differences in rates of pre-existing respiratory pathology, Mallampati Score, Cormack and Lehane’s classification between complications and no-complications groups (Fig. 2c-e). Lastly, all the patienta were able to be masked.

**Fig. 3:**
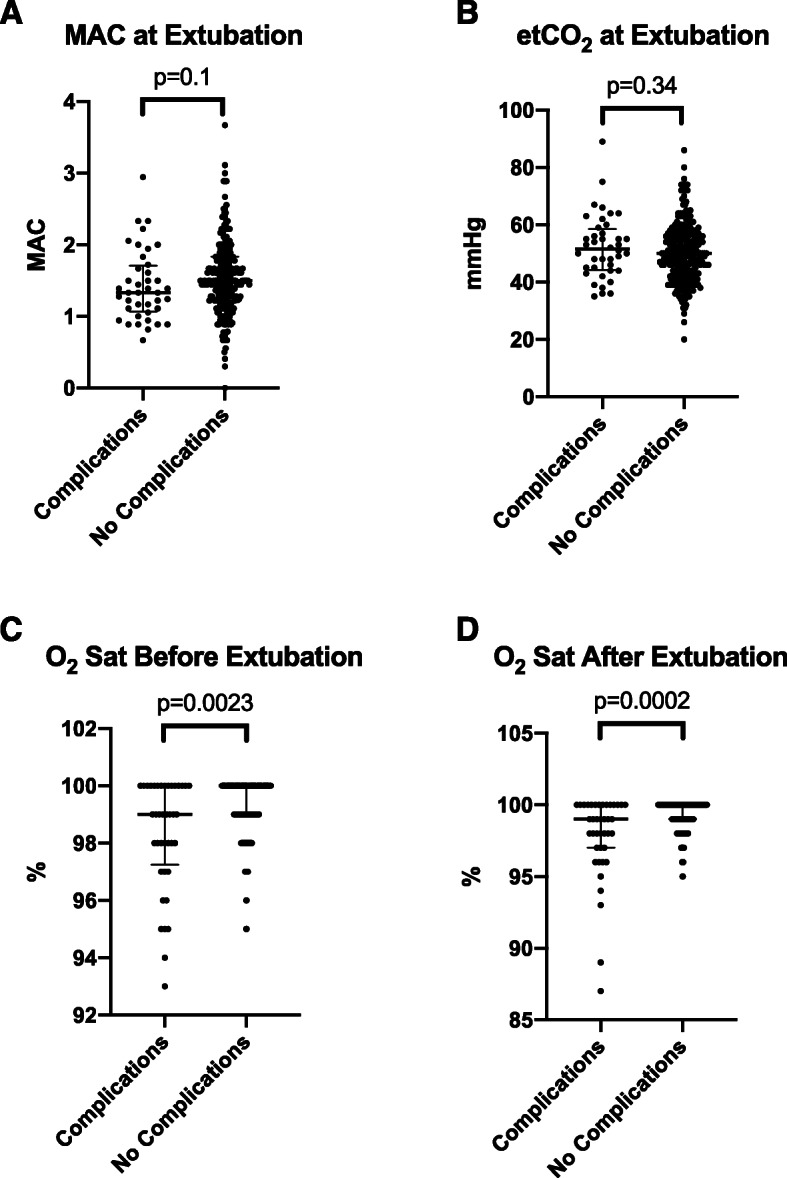
Comparison of emergence conditions between complications versus no complications groups by **a** MAC, **b** end-tidal CO_2_ (etCO_2_), (C) O_2_ Saturation (Sat) before and (D) O_2_ Sat after extubation

Anesthetic depth did not appear to impact complications at the time of extubation MAC (1.33(1.07–1.71) vs 1.50(1.22–1.83, *p* = 0.1002), nor did etCO_2_ (51.5(44.3–58.5) vs 50.0 (43.0–57.0), *p* = 0.3352) (Fig. 3a & b). However, patient percent O_2_ saturation levels are significantly lower for the complication group compared to the no complications group at 5 mins before deep extubation (99.0(97.3–100) vs 100 (99.0–100), *p* = 0.0023) (Fig. 3c).

**Fig. 4:**
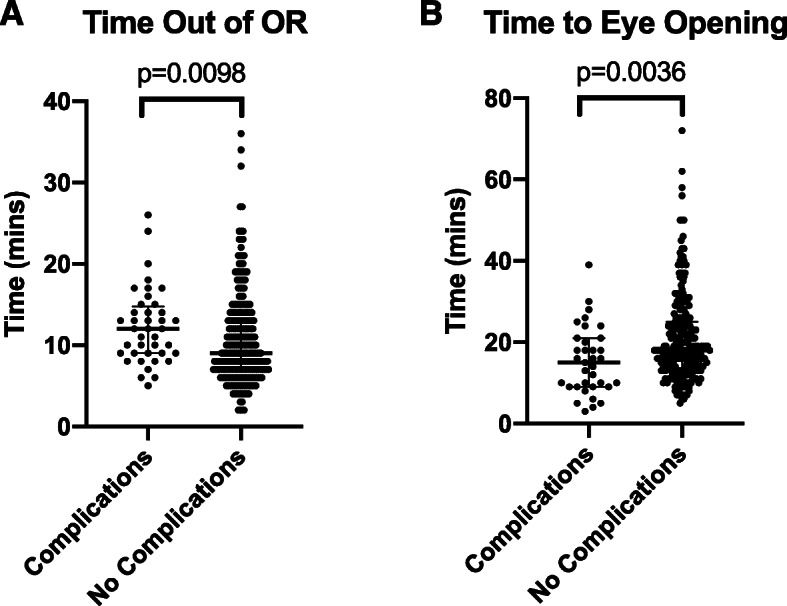
Comparison of emergence times between complications versus no complications groups from end of surgery to **a** time out of OR and from extubation to **b** time to eye opening

The time from deep extubation to leaving the OR was longer, at 12.0(9.00–14.8) mins, in the complications group compared to 9.00(7.00–13.0) mins in the no complications group (*p* = 0.0098) (Fig. 4a). The time to eye opening was also longer in the complications group than the no complications group (15.0(9.00–21.0) vs 18.0(13.3–25.0), *p* = 0.0036) (Fig. 4b). The total intraoperative opioid use and muscle relaxant and reversal use are not significantly different between the two groups (Table 1).

**Table 1:** Comparison of intraoperative dose of medications. Drug name (dosing unit) are listed in the left column. Data are expressed as median (q1-q3)

Drugs	Complications (***n*** = 40)	No Complications (***n*** = 260)	***P***-Value
Fentanyl (mcg)	100 (0.0–100.0)	100 (0.0–100.0)	0.3674
Remifentanil (mg)	0.580 (0.15–0.973)	0.435 (0.100–0.960)	0.3133
Morphine (mg)	0.0 (0.0–2.0)	0.0 (0.0–2.0)	> 0.9999
Hydromorphone (mg)	0.200 (0.00–0.900)	0.00 (0.00–0.500)	0.3374
Rocuronium (mg)	10.0 (0.00–10.0)	10.0 (0.00–10.0)	0.5999
Succinylcholine (mg)	100 (0.00–100)	80.0 (0.00–100)	0.6332
Neostigmine (mg)	0.00 (0.00–0.00)	0.00 (0.00–0.00)	0.5735

## Discussion

In this study, 13% of adult patients (40 out of 300) had at least one or more respiratory complications with deep extubation. This is within range of a previous publication by Kim and colleagues in which one group that received desflurane had a 48% complications rate (12 out of 25 patient) while the other group that received desflurane with remifentanil had a 3.4% complication rate (1 out of 29 patients) [10]. It is also consistent with Fan et al’s report, where percentage of patient with airway complications ranges from 12 to 37.5% [11]. An important difference between ours and prior studies is how respiratory complications are defined. For example, whereas Kim et al’s defined complications as coughing and breath holding, we expanded the criteria to capture additional complications, including significant desaturation, laryngospasm, stridor, bronchospasm and reintubation, that could also influence the success of deep extubation. It is worth noting that all of these complications were easily corrected by the anesthesia providers in our study with no need for drastic interventions such as reintubation. However, our data also showed that patients who had complications with deep extubation tended to stay longer in the OR compared with patients who did not.

It is well understood that deep extubation can minimize adverse hemodynamic reflexes in appropriate situations [12]. Nonetheless, many anesthesiologists are reluctant to perform deep extubation in adults because of concerns for potential respiratory complications [5]. The present study indicates that deep extubations in adults is likely safer than in the pediatric population. Our airway complication rate of 13% in adult patients is significantly lower than the 40% complication rate (64 out of 159 patients) reported in a recent meta-analysis of pediatric patients [13]. While it is possible that patient selection and provider difference account for the lower rate; it is also conceivable that the pediatric airway is more irritable and sensitive to stimulation than the adult airway [14].

Present study suggests that patient selection plays an integral part in the success of endotracheal deep extubations. Our anesthesia providers selected patients for deep extubations per clinical discretion without pre-determined criterion. Overwhelmingly, the patients selected had easy airway placement based on the Cormack and Lehane’s Grade as only 1 patient out of 300 had a grade 4 view, which is a probable factor contributing to an overall complications rate near the lower limits of previously published ranges [10, 11]. On the flip side, our data also shows that when the provider chose to deep extubate patient with lower O_2_ saturation levels 5 mins prior to extubation, these patients are more likely to have significant airway complications. Our results suggest that higher BMI patients are less likely to tolerate deep extubations. We observed a statistically significant correlation between higher BMI and likelihood of complications during deep extubation. The median BMI in the complications group was 30 while the median BMI in the no complications group was 26. Obesity has been shown to worsen oxygenation through several mechanisms, including increased intraabdominal pressure and atelectasis [15–17]. Whether an isolated elevated BMI is a causal factor for complications during deep extubations will need further investigation.

The depth of anesthesia suitable for a smooth deep extubation is primarily based on the MAC of inhaled anesthetics. Previous studies suggested that extubation could be performed at an inhaled anesthetic level as low as 1 MAC [2, 11, 18–20]. Some of the differences in MAC levels were likely due to variations in adjuvant opioid use, because opioid medications have been shown to minimize coughing and various extubation related adverse events [21, 22]. Here, we allowed the providers to freely decide the type and amount of opioid use appropriate for practice and did not observe a significant difference in the amount of opioid used in the complications versus no complications groups.

There were several limitations to this study. Firstly, this is a single-center prospective study, and the anesthesiologists were not and could not be blinded to the treatment technique. Secondly, there is also significant selection bias in the study, as no patients with history of difficult airway underwent deep extubation. Thirdly, other than the deep extubation technique, the anesthetic management was not standardized. However, this is a reality of every day anesthesia practice, irrespective of the extubation technique. Lastly, an experienced anesthesia provider remained with each patient until an adequate control of the airway was achieved, which could have contributed to the low incidence rate of complications. Moving forward, we hope our data can facilitate a more informed calculation of sample size for future studies comparing the complication rate of deep versus awake extubation in adults. As expected, time to leaving the OR was higher in the complication group, however, the general question about differences in operating room turnover times between deep and traditional extubation techniques is beyond the scope of this study. Finally, there are probably many different ways of performing a deep extubation and further studies should be done to fine tune this technique.

## Conclusions

Our findings demonstrate that deep extubation in adults is associated with a relatively low complication rate. Furthermore, high BMI and low O_2_ saturation levels pre-extubation are associated with increased complications. We acknowledge that deep extubation should not be performed in patients with a known of history of difficult airway or aspiration risk and should always be performed by experienced providers after careful assessment. However, our experience does support deep extubation as a feasible and safe option in appropriate clinical circumstances.

## Data Availability

The datasets used and/or analyzed during the current study available from the corresponding author on reasonable request.

## Abbreviations

ASA PS: American Society of Anesthesiologists Physical Status
BMI: Body Mass Index
FiO2: Fraction of inspired Oxygen
GA: General Anesthesia
IRB: Institutional Review Board
MAC: Minimum Alveolar Concentration
MEEI: Massachusetts Eye and Ear Infirmary
PACU: Post Anesthesia Care Unit
SPO_2_: Saturation of Peripheral Oxygen
